# Diagnostic Tools for Endometriosis in Poland: A Comparative Assessment of Reliability and Out-of-Pocket Costs

**DOI:** 10.3390/jcm14144935

**Published:** 2025-07-11

**Authors:** Anna Rogalska, Katarzyna Brukało

**Affiliations:** 1 Department of Health Economics and Health Management, Faculty of Public Health in Bytom, Medical University of Silesia, 41-902 Bytom, Poland; arogalska@sum.edu.pl; 2 Department of Health Policy, Faculty of Public Health in Bytom, Medical University of Silesia, 41-902 Bytom, Poland

**Keywords:** endometriosis diagnostics, cost analysis, patient perspective, Poland

## Abstract

**Objectives:** This study aimed to assess the availability, diagnostic reliability, and out-of-pocket costs of endometriosis diagnostic tools available on the private healthcare market in Poland. **Methods:** A desk-based analysis was conducted from a patient perspective to identify commercially available diagnostic tests for endometriosis in Poland. Data were collected in September 2024 using relevant keywords to simulate a patient search process. Identified tests were evaluated for their compliance with the 2022 European Society of Human Reproduction and Embryology (ESHRE) guidelines. Key parameters, including sensitivity, specificity, and associated costs, were assessed based on the available literature. Out-of-pocket costs were compared between the private and public healthcare sectors. **Results:** Five diagnostic methods were identified in the private healthcare market: two imaging techniques (transvaginal ultrasound, magnetic resonance imaging) and three blood-based tests. None of the blood-based tests demonstrated sensitivity or specificity above 90%. Imaging techniques met this criterion. The cost of blood tests ranged from EUR 21.1 to EUR 467.77. The average private-sector cost for transvaginal ultrasound was EUR 111.64, representing a 482.6% increase compared to the public sector. Magnetic resonance imaging cost EUR 122.89 in the private sector, a 148.64% increase. **Conclusions:** The private Polish healthcare market lacks non-invasive diagnostic tests for endometriosis that achieve high reliability based on large study samples. Imaging tests, while reliable, pose significant financial barriers when accessed privately. Enhanced public access and clearer patient guidance are required to ensure timely and effective diagnosis.

## 1. Introduction

Endometriosis is a chronic inflammatory disease [1], now considered a systemic disease rather than a disease predominantly affecting the pelvis. This disease affects metabolism in the liver and adipose tissue, leads to systemic inflammation, alters gene expression in the brain, which causes pain sensitization and mood disorders [2], and has significant implications for a woman’s fertility [3,4] and quality of life, including work and social relationships. It also poses an economic burden because the disease can lead to the loss of productivity at work as well as high utilization of health resources [5,6]. As shown by the results of a systematic review, the economic burden of endometriosis is significant, taking into account both direct healthcare costs and productivity losses. The mean annual costs per woman ranged from EUR 8768 in Sweden to USD 16,970–20,898 in Australia [7]. Unfortunately, there are no studies estimating the economic burden of endometriosis in Poland.

The clinical manifestations of this condition are nonspecific, ranging from asymptomatic to severe dysmenorrhea, profound dyspareunia, and chronic pelvic pain, as well as intestinal and bladder symptoms and fatigue. However, the severity of symptoms is not correlated with the degree of disease [8,9]. The etiology and pathogenesis of endometriosis are unclear, and there is a lack of effective treatment measures [10]. Nonetheless, some studies suggest that endometriosis may be associated with increased numbers of Proteobacteria, Enterobacteriaceae, Streptococcus, and Escherichia coli in different parts of the microbiome [11], suggesting a key role for changes in mitochondrial function in many cell types and body systems, including the immune system [3].

It is estimated that endometriosis affects approximately 10% of the general female population in the world [12]. Endometriosis can be divided into three different types: superficial peritoneal endometriosis (SPE), ovarian endometrioma (OMA), and deep infiltrating endometriosis (DIE). Most international guidelines now recommend a nonsurgical diagnosis based on symptoms and findings on physical examinations and imaging [8]. It sometimes happens that endometriosis is diagnosed accidentally during surgery performed for other indications when the patient does not report any symptoms. The differential diagnosis of dysmenorrhea in endometriosis can be distinguished from primary dysmenorrhea, which has a shorter duration (<72 h) and responds well to nonsteroidal anti-inflammatory drugs [8]. Moreover, the World Endometriosis Society consensus recommends that endometriosis diagnosis and management should be incorporated into primary healthcare, offering patients individualized care over a long-term period [13]. Some researchers suggest that pre-diagnosis all-cause and endometriosis-related healthcare costs were higher among patients with longer diagnostic delays [14]. Others say that the highest resource utilization and costs experienced by endometriosis patients occur in the first year after diagnosis [15].

According to the ESHRE guidelines, clinicians are recommended to use imaging (ultrasound (US) or MRI) in the diagnostic work-up for endometriosis, but they need to be aware that a negative finding does not exclude endometriosis. And also, practitioners should not use measurements of biomarkers in endometrial tissue, blood, menstrual, or uterine fluids to diagnose endometriosis [16]. Imaging techniques have limited ability to detect superficial endometriosis [6].

Artificial intelligence (AI) and its subset, machine learning (ML), are increasingly being used in laboratory medicine to minimize subjectivity in test interpretation and ultimately improve patient care. One test for endometriosis that uses AI is the zwingEndotest [17].

Due to the limitations present in the Polish public services market, which do not result from the lack of access to diagnostics recommended by European guidelines (such as transvaginal ultrasound—TVS or magnetic resonance imaging—MRI) in the diagnosis of endometriosis, but from the lack of specialized reference centers within the public healthcare system and the lack of appropriate information on locations where specialists with the skills to diagnose this disease are available, patients are often forced to seek diagnostics on the private market, incurring costs out of their own pockets. In connection with the above, this study aimed to analyze diagnostic tests for endometriosis available in the Polish commercial market. Additionally, the aim was to estimate the costs incurred by the patient out of pocket.

## 2. Materials and Methods

In September 2024, endometriosis diagnostic methods available on the Polish market were searched. To identify diagnostic tests, the patient’s perspective was adopted, and available tests were searched on the Polish commercial market using the following keywords: endometriosis diagnostics, endometriosis tests, and endometriosis testing methods. The available diagnostic tests for endometriosis in the private market were assessed, and they are recommended on the ESHRE Guideline Endometriosis: https://www.eshre.eu/Guidelines-and-Legal/Guidelines/Endometriosis-guideline (accessed on 15 September 2024). In the next step, we will take into account their sensitivity, specificity, and costs. Data on the sensitivity and specificity of diagnostic tests were obtained from the literature, and the size of the study groups was also taken into account. To estimate the out-of-pocket costs incurred directly by patients, the prices of diagnostic methods were presented as average prices for private TVUS in 7 cities, and for MRI recommended for endometriosis in 2 cities across the country and compared with the prices on the public services market in 2024.

Cost values in the Polish currency (PLN) were converted into euros according to data from the National Bank of Poland as of 20 September 2024—1Euro = PLN 4.28.

## 3. Results

**Figure 1:**
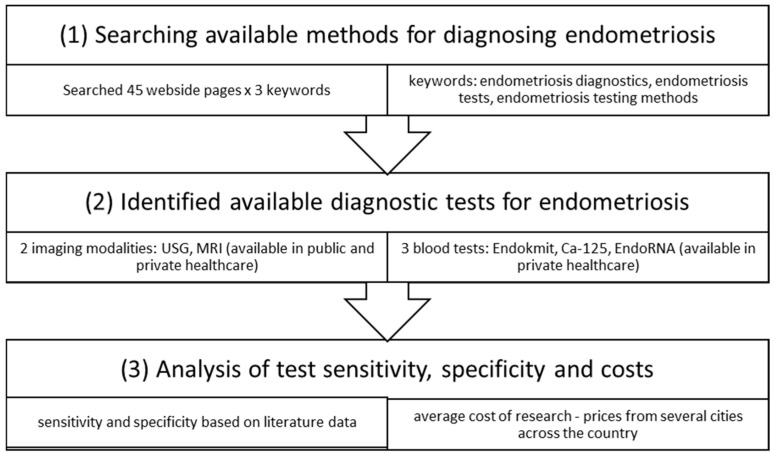
Stages of research implementation.

The diagram below presents the stages of implementation of this study (Figure 1).

After analyzing the available diagnostic tests for three blood tests, none had a sensitivity or specificity of at least 90%. Their prices ranged from EUR 21 to EUR 467.77, of which in the case of the two most expensive diagnostic tests, the sensitivity and specificity were tested on small groups (Table 1).

**Figure 2:**
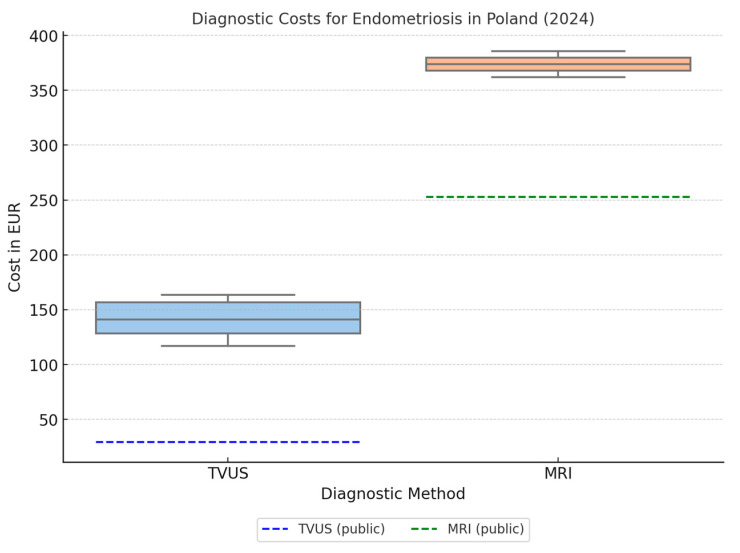
Distribution of private out-of-pocket costs (EUR) for transvaginal ultrasound (TVUS)—blue box and magnetic resonance imaging (MRI)—Orange box, for endometriosis diagnostics in Poland (2024). Boxplots illustrate the interquartile range, median, and variability across seven cities. Dashed lines indicate the average costs in the public healthcare system for TVUS (29.18 EUR) and MRI (252.63 EUR).

Due to the lack of an available non-invasive endometriosis diagnostic method that meets the criteria of >90% sensitivity and specificity, cost-effectiveness analysis compared to available diagnostic methods could not be performed (Table 2). Two diagnostic methods with ≥90% sensitivity and specificity are available in both public and private markets; these are imaging methods: ultrasound and pelvic magnetic resonance imaging (recommended by European guidelines, and Polish guidelines [21]). The average cost of a TVUS examination for private patients was EUR 111.64 (482.6%) higher than within the public healthcare system. The cost of an MRI examination, in contrast, in the private market was higher by EUR 122.89 (148.64%) compared to an examination in the public system (Figure 2). Although not included in the figure, the Endotest—a private laboratory test—is offered at a significantly higher cost (~535 EUR).

## 4. Discussion

Diagnostic tests in patient care settings must be evidence-based [21] to make clinical decisions and guide patient care. Diagnostic procedures are of high quality if they meet the conditions of safety, timeliness, efficiency, effectiveness, patient-centeredness, and ethicality. A trial is of high value if the clinical benefits are significantly greater than the risks and costs of the trial [23]. In this study, five available diagnostic tests for endometriosis were identified in the Polish private medical services market, of which 3/5 had sensitivity and specificity that failed to exceed 90%, with the cheapest costing EUR 21 and the most expensive one costing EUR 477. Both the long search for a pain diagnosis, often described in the medical literature, and the lack of full information about the accuracy of diagnostic tests for endometriosis can lead to unnecessary costs for patients. Therefore, patients need access to full and reliable data on the diagnostic effectiveness of tests.

When analyzing the costs of available tests, it is worth considering the specific nature of the disease, endometriosis—European guidelines do not recommend pharmacological and/or surgical treatment due to the very fact of endometriosis diagnosis but due to the health problems associated with it. On the other hand, diagnosing endometriosis, merely confirming its presence or absence, is insufficient to initiate treatment. Aspects such as the subtype, location, and extent of the disease are important factors considered in clinical management [4]. Because deep endometriosis can cause the capacity to cause end-organ damage, such as kidney failure (from ureteric obstruction) or bowel obstruction, timely diagnosis and management are important [8]. In the literature, you can find an analysis of the test for diagnosing endometriosis from saliva, using the Endotest test. Despite the high sensitivity and specificity of the Endotest diagnostic test, information about a positive/negative endometriosis result seems to be insufficient to implement further treatment. In addition, there is a saliva diagnostic test (Endotest^®^) based on the saliva miRNA signature, using a combination of next-generation sequencing (NGS) and artificial intelligence. A prospective ENDO-miRNA study including saliva samples collected from 200 women with chronic pelvic pain shows that it showed high sensitivity and specificity [24], but was conducted on a small study group. The small sample size means that there may be bias due to a lack of representativeness, and the results may be subject to the risk of being overly optimistic.

It is worth emphasizing that although TVUS and MRI tests are characterized by high sensitivity and specificity for endometriosis diagnosis, they concern the type of endometriosis localized in the ovary. TVUS shows moderate sensitivity scores for deep endometriosis, with the sonographic evaluation of superficial endometriosis still in its infancy [25]. Moreover, other factors such as the social normalization of women’s pain may contribute to prolonging the time to diagnosis [26,27]. Another important aspect when comparing available diagnostic imaging methods for endometriosis is the need for training and skills in this field. Despite good evidence for the accuracy of ultrasound, its wide availability and the lack of contraindications to its use, it is often not a diagnostic modality due to, among other things, the lack of training and skills in this field, which is characterized by a learning curve, i.e., improvement in the performance of a given task. In the case of ultrasound, this would include not only acquiring theoretical knowledge and its application in pattern recognition, but also learning how to manipulate the probe, which requires good hand-eye coordination and manual dexterity. In the case of MRI, on the other hand, the learning curve may be shorter because manual dexterity is not necessary [28].

Patients need to be comprehensively informed about the importance of the sensitivity and specificity of diagnostic methods, which will allow them to make informed decisions based on reliable medical data. The decision to choose diagnostics should not be based solely on cost, but also on understanding how the precision of tests affects the accuracy of diagnosis and appropriate therapeutic management. Unfortunately, no studies have compared the sensitivity and specificity of endometriosis detection using TVUS and MRI in the case of routine gynecological TVUS examination and TVUS examination performed by gynecologists reporting on these skills. As reported by VanBuren et al., the spectrum of abnormalities associated with endometriosis diagnosis requires a pattern-based approach that includes diagnostics using specialized ultrasound and MRI protocols [29].

Although TVUS in the private system is 450% more expensive than in the public system, it is the most cost-effective method of the three strategies analyzed. However, this is an out-of-pocket cost for the patient. Due to the shortage of specialists with skills in diagnosing endometriosis on the public market and the simultaneous increased demand for these services, despite the long waiting time for an appointment as part of the private service (about a year), the price of a diagnostic test is constantly increasing. This situation exposes patients to higher out-of-pocket expenses to obtain a diagnosis, which in turn deepens the problem of health inequalities due to costs, requiring in-depth analysis. The limitations of endometriosis imaging suggest the potential of artificial intelligence (AI) to improve diagnostics. AI algorithms are trained to detect endometriosis on ultrasound. As presented in a 2025 systematic review by Mittal et al., neural network and deep learning methods can classify the presence of endometriosis from ultrasound data. However, results from a larger validation study are pending [30].

A limitation of this study is the difficulty in accessing data on sensitivity and specificity by the location of endometriosis (localization on the ovaries is easier during transvaginal ultrasound examination compared to peritoneal endometriosis and deeply infiltrating endometriosis). Another limitation may be that this study focused only on information from one country.

## 5. Conclusions

Our analysis revealed that no blood- or saliva-based diagnostic tests currently available on the Polish market meet the criteria of both high sensitivity and specificity (above 90%) and validation in large study populations. This makes it impossible to perform a reliable cost-effectiveness analysis for these tests at this stage.

Meanwhile, the high cost of imaging diagnostics in the private sector can pose a significant financial barrier for patients, potentially contributing to diagnostic delays or missed diagnoses altogether.

Addressing the challenges in endometriosis diagnostics requires a multidimensional approach. It is recommended that specialist training in advanced TVUS-based protocols be introduced into the curricula of medical students pursuing gynecology.

Further research is warranted to

1. Assess the real-world effectiveness of diagnostic tools used in both public and private healthcare in Poland;
2. Monitor the implementation of novel non-invasive technologies;
3. Regularly evaluate and adapt diagnostic pathways in line with technological advancements.

To date, no studies have systematically compared the effectiveness of imaging methods such as TVUS and MRI across the public and private sectors in Poland. This remains an important area for future investigation to ensure equitable and effective access to accurate diagnosis.

## Figures and Tables

**Table 1:** Cost of endometriosis diagnostic tests available on the Polish market with ≤90% specificity and sensitivity (as of 20 September 2024).

Tests	Costs (Euro)	Specificity (%; n)	Sensitivity (%; n)	ESHRE Guideline Endometriosis
EndomKIT (CA125, BDNF)	104.82	95.6%; n = 204 [15]	46.2%; n = 204 [18]	-
Ca-125	21.1	92.7%; n = 2920 [16]	52.4%; n = 2920 [19]	not recommended to diagnose endometriosis.
EndoRNA qRT-PCR test	467.77	89.29%; n = 77 [17]	94.12%; n = 77 [20]	-

**Table 2:** Endometriosis diagnostic methods with ≥90% sensitivity and ≥90% specificity.

Methods of Diagnosing Endometriosis	Specificity (%; n)	Sensitivity (%; n)	Methods of Diagnosing Endometriosis	Specificity (%; n)
Transvaginal ultrasound	95%; n = 1976 [22] systematic review	91% [22] systematic review	Transvaginal ultrasound	95%; n = 1976 [18] systematic review
Magnetic resonance imaging	94%; n = 1976 [21] systematic review	91–93.5% [22] systematic review	Magnetic resonance imaging	94%; n = 1976 [18] systematic review

## Data Availability

The original contributions presented in this study are included in the article. Further inquiries can be directed at the corresponding author.

## Abbreviations

The following abbreviations are used in this manuscript:

TVUS	Transvaginal Ultrasound
MRI	Magnetic Resonance Imaging
ESHRE	European Society of Human Reproduction and Embryology
